# Genome Sequence of *Paracoccus* sp. JM45, a Bacterial Strain Isolated from a Marine Sponge with a Dual Quorum Sensing Inhibition Activity

**DOI:** 10.1128/MRA.01496-18

**Published:** 2019-01-10

**Authors:** José A. Gutiérrez-Barranquero, María L. Parages, Alan D. W. Dobson, F. Jerry Reen, Fergal O’Gara

**Affiliations:** a BIOMERIT Research Centre, School of Microbiology, University College Cork, Cork, Ireland; b School of Microbiology, University College Cork, Cork, Ireland; c Environmental Research Institute, University College Cork, Cork, Ireland; d Telethon Kids Institute, Perth, Western Australia, Australia; e Human Microbiome Programme, School of Pharmacy and Biomedical Sciences, Curtin Health Innovation Research Institute, Curtin University, Perth, Western Australia, Australia; University of Rochester School of Medicine and Dentistry

## Abstract

The draft genome sequence of *Paracoccus* sp. strain JM45, isolated from a marine sponge harvested off the west coast of Ireland, is reported here. Quorum sensing and quorum sensing inhibition activities have been reported recently for this bacterium, and genomic analysis supports its potential use for novel therapeutic development.

## ANNOUNCEMENT

Paracoccus is a Gram-negative bacterial genus belonging to the *Alphaproteobacteria* class. Paracoccus denitrificans is the best-characterized member of this genus, and this is due in part to its broad metabolic diversity (1), capacity for denitrification (2), and ability to degrade organic compounds (3). Recently, the discovery of the cell-cell signaling phenomenon referred to as quorum sensing (QS) in Paracoccus denitrificans captured the attention of many researchers, and QS has been reported to be involved in biofilm formation, iron uptake, and denitrification in this organism (4, 5)*. Paracoccus* sp. strain JM45 was isolated from a marine sponge sample belonging to the genus *Polymastia* collected off the west coast of Ireland as part of the marine biodiscovery cruise performed in May 2010. To isolate *Paracoccus* sp. strain JM45 from a marine sponge, we followed a protocol previously described with minor modifications in the use of SYP-SW (soluble starch, yeast extract, peptone-seawater) medium and marine agar (Difco) (6). We previously reported QS and quorum sensing inhibition (QSI) properties for this bacterial strain (7). Therefore, here, we announce the genome sequence of *Paracoccus* sp. strain JM45, a promising source of novel QSI compounds with potential for controlling multidrug-resistant pathogens. Total DNA of *Paracoccus* sp. strain JM45 was extracted using the UltraClean microbial DNA isolation kit (Mo Bio Laboratories, Inc., Carlsbad, CA) and was subjected to DNA library preparation using a TruSeq exome library prep kit. The draft genome sequencing project of *Paracoccus* sp. strain JM45 was performed by the Beijing Genomics Institute (BGI, China) using the Illumina HiSeq 4000 sequencing platform involving paired-end reads with a read length of 150 bp. The superfast FASTA/Q file manipulation tool, readfq.v5 (BGI unpublished software [8]), was used for quality trimming. This software removes the paired-end reads with a certain proportion of low-quality bases (default, 40%; parameter setting, 6 bp), reads with a certain proportion of Ns (ambiguous bases; default, 10%; parameter setting, 10 bp), reads with adapter contamination (default, 15 bp overlapped between adapter and reads), and duplicate sequences. Thus, the high-quality-filtered reads were all 150 bp long. A total of 166.66 Mb of data were generated, and high-quality reads were assembled using SOAPdenovo 2.04 with default parameters. The sequencing depth provided 45× coverage of the genome. The draft genome assembly comprised 84 contigs with an *N*_50_ value of 167,336 grouped into 81 scaffolds with a total size of 3,602,847 bp and an overall GC content of 59.1%. Genome sequence annotation and gene identification were carried out by the Rapid Annotations using Subsystems Technology (RAST) server version 2.0 using default parameters and the RAST tool kit (RASTtk) for annotation pipelines (9, 10) and by the NCBI Prokaryotic Genome Annotation Pipeline (PGAP) using default parameters. Detection of secondary metabolite gene clusters was performed using antiSMASH bacterial version 3.0 (11). Based on PGAP annotation results, 3,516 coding sequences, 3 ribosomal RNAs, and 45 tRNAs were detected. Two potential acyl homoserine lactone (AHL)-based quorum sensing systems were found in scaffolds 7 and 18. Both systems are related to the production of long-chain AHLs, consistent with our previous finding of AHL-based QS activity in this isolate (7), which was confirmed using the Agrobacterium tumefaciens NTL4 biosensor strain (12). In the context of QSI properties, a penicillin amidase enzyme with potential for AHL degradation was encoded in scaffold 1. Finally, antiSMASH predicted several potential gene clusters involved in secondary metabolite production. A gene cluster observed and related to the production of a novel polyketide by type III polyketide synthases was of particular interest. Polyketides are an important class of biologically active compound (13), and while further *in vitro* investigations are required to establish activity profiles, *in silico* identification through antiSMASH supports the bioactive potential of *Paracoccus* sp. strain JM45. Furthermore, an ectoine biosynthetic gene cluster was found in scaffold 1. Based on known activities of ectoine and other compatible solutes, this could be relevant for growth at high concentrations of salt, as previously demonstrated in other bacteria (14).

### Data availability.

This whole-genome shotgun project has been deposited at DDBJ/ENA/GenBank under the accession number QQVY00000000. The version described in this paper is version QQVY01000000. The raw reads from this study have been submitted to the NCBI Sequence Read Archive (SRA) under the accession number SRP166726.
